# Ceramide Nanoliposomes as Potential Therapeutic Reagents for Asthma

**DOI:** 10.3390/cells12040591

**Published:** 2023-02-11

**Authors:** Harumi Sakae, Yuri Ogiso, Masaya Matsuda, Hayato Shimora, Tye Deering, Todd E. Fox, Mark Kester, Takeshi Nabe, Kazuyuki Kitatani

**Affiliations:** 1 Laboratory of Immunopharmacology, Faculty of Pharmaceutical Sciences, Setsunan University, Hirakata 573-0101, Japan; 2 Department of Pharmacology, University of Virginia, Charlottesville, VA 22908-8735, USA

**Keywords:** ceramide nanoliposome, ceramide, asthma, inflammation, goblet cell, EGF

## Abstract

Ceramides are an emerging class of anti-inflammatory lipids, and nanoscale ceramide-delivery systems are potential therapeutic strategies for inflammatory diseases. This study investigated the therapeutic effects of ceramide nanoliposomes (CNL) on type 2 inflammation-based asthma, induced by repeated ovalbumin (OVA) challenges. Asthmatic mice intratracheally treated with ceramide-free liposomes (Ghost) displayed typical airway remodeling including mucosal accumulation and subepithelial fibrosis, whereas, in CNL-treated mice, the degree of airway remodeling was significantly decreased. Compared to the Ghost group, CNL treatment unexpectedly failed to significantly influence formation of type 2 cytokines, including IL-5 and IL-13, known to facilitate pathogenic production of airway mucus predominantly comprising MUC5AC mucin. Interestingly, CNL treatment suppressed OVA-evoked hyperplasia of MUC5AC-generating goblet cells in the airways. This suggests that CNL suppressed goblet cell hyperplasia and airway mucosal accumulation independently of type 2 cytokine formation. Mechanistically, CNL treatment suppressed cell growth and EGF-induced activation of Akt, but not ERK1/2, in a human lung epithelial cell culture system recapitulating airway goblet cell hyperplasia. Taken together, CNL is suggested to have therapeutic effects on airway remodeling in allergic asthma by targeting goblet cell hyperplasia. These findings raise the potential of ceramide-based therapies for airway diseases, such as asthma.

## 1. Introduction

Asthma is a chronic inflammatory disease characterized by inflammation-associated airway remodeling and hyperresponsiveness [1,2]. Asthma pathogenesis is associated with type 2 inflammation together with key effecter cells, such as T-helper 2 (Th2) cells, dendritic cells, group 2 innate lymphoid cells (ILC2s), airway epithelial cells, and eosinophils [3]. These cells contribute to the multiple features of allergic inflammation by secreting a myriad of proinflammatory mediators that deteriorate vasodilation, vascular permeability, airway smooth muscle contraction, mucus secretion, and immune cell recruitment [4]. Thus, given the important roles of type 2 cytokines such as interleukin (IL)-4, IL-5, and IL-13, in pathogenesis, they are attractive therapeutic targets [2,3,5].

Asthma is caused both by environmental and genetic factors. Genome-wide association studies identified multiple single nucleotide polymorphisms in 17q21 that account for non-allergic childhood asthma along with increased orosomucoid-like protein 3 (ORMDL3) expression as a principal genetic determinant [6]. ORMDL3 [7,8] is an inhibitory protein for serine palmitoyl CoA transferase that catalyzes the condensation of serine and palmitoyl CoA in the first step of de novo sphingolipid synthesis. In recent clinical studies, the 17q21 locus polymorphism was demonstrated to associate with decreased activity in de novo sphingolipid synthesis and whole blood sphingolipids (dihydroceramides, ceramides, sphingomyelins), but not plasma sphingolipids [9]. Therefore, dysregulated sphingolipid synthesis is a possible factor in asthma pathogenesis and sphingolipid modulation is a potential asthma therapeutic strategy.

Sphingolipids are prerequisites for the formation and integrity of cellular biomembranes and lipid rafts and in controlling cellular behaviors including regulated cell death and proinflammatory responses [10,11,12,13,14]. Ceramide, a central molecule in sphingolipid metabolism, is formed by multiple pathways, such as de novo, salvage, and sphingomyelinase pathway [15]. Importantly, ceramides are proposed inhibitory molecules for proinflammatory responses [16,17,18]. The cell-permeable short-chain C_6_-ceramide suppresses FcεRI-mediated activation of protein kinase C, extracellular signal-regulated kinase (ERK) 1/2, p38, and cytosolic phospholipase A_2_ in mast cells [16,19]. Izawa et al. demonstrated that ceramides act as a ligand for leukocyte mono-immunoglobulin-like receptor 3 (LMIR3)/CD300f and inhibit FcεRI-mediated mast cell activation [20]. Moreover, in phorbol ester-induced cellular proinflammatory responses of epithelial cells, C_6_-ceramide inhibited p38 activation and p38δ-mediated production of IL-6 by activating serine/threonine protein phosphatases [17,18]. Thus, ceramides are believed to preferentially target and inhibit cellular proinflammatory responses. These features are instrumental in developing ceramide-based therapies for inflammatory diseases.

Given the bioactivities of ceramides, proposed ceramide-based therapies for inflammatory diseases exist [18,21]. In a murine model of corneal inflammation, in vivo treatment with liposomal C_6_-ceramide suppressed neutrophil infiltration to the corneal stroma and resultant corneal haze induced by lipopolysaccharide or *S. aureus*. Mechanistically, liposomal ceramides were revealed to suppress activation of c-Jun N-terminal kinase (JNK) and p38 and production of neutrophil chemotactic cytokines such as CXCL1, CXCL5, and CXCL8 in corneal epithelial cells [21]. Therefore, ceramide-based therapies may be effective approaches for inflammatory disease treatments.

Nanoscale formulations are shown to dramatically improve the pharmacokinetic and toxicological profiles of ceramide delivery to cells [22,23,24]. We developed non-toxic and biologically stable nanoliposomes of C_6_-ceramide (referred to as ceramide nanoliposomes, CNL) whose therapeutic efficacy for suppressing cancer progression is validated by multiple preclinical studies [22,23,25] supporting an FDA phase 1 first-in-man dose escalation study.

The majority of asthmatic patients are controlled by anti-inflammatory/bronchodilating agents, but those with severe asthma respond poorly to the conventional therapy [26,27]. A growing need to find a novel target for asthma is increasing and CNL could be repositioned as therapeutic reagents for asthma. In this study, we provide evidence supporting CNL as a novel reagent for asthma therapy.

## 2. Materials and Methods

### 2.1. Materials

MUC5AC antibody (sc-16903) was purchased from Santa Cruz Biotechnology (Dallas, TX, USA). Rabbit antibodies specific for phospho-p38 (#9215S), p38α (#9218), phospho-JNK1/2 (#4668S), JNK1/2 (#9252S), phospho-ERK1/2 (#4370), ERK1/2 (#9102), phospho-protein kinase B (Akt) Ser473 (#9271S), phospho-Akt Thr308 (#2965S) and Akt (#9272S) were obtained from Cell Signaling Technology (Danvers, MA, USA). Tissue protein extraction reagents (T-PER), SuperSignal West Dura Extended Duration Substrate, collagenase type I, allophycocyanin-conjugated anti-mouse IL-33 receptor (ST2) antibody (17-9335-82), eFluor660-conjugated anti-mouse Foxp3 antibody (50-5773-82), Fast SYBR™ Green Master Mix, Fixation/Permeabilization Concentrate, Pierce™ BCA Protein Assay Kit, enzyme-linked immunosorbent assay (ELISA) kits for IL-4, IL-5, and IL-13, and mouse antibodies specific for β-actin were purchased from Thermo Fisher Scientific (Waltham, MA, USA). Horseradish peroxidase (HRP)-conjugated antibodies for mouse and rabbit IgG were purchased from Jackson ImmunoResearch (West Grove, PA, USA). High glucose Dulbecco’s modified Eagle’s medium (DMEM), HistoVT one, deionized and sterilized water, and trypsin were purchased from Nacalai Tesque (Kyoto, Japan). Fetal bovine serum (FBS) was purchased from Biowest (Nuaillé, France). Grade V ovalbumin (OVA, purity of ≥98%), monensin sodium salt, paraformaldehyde, and anti β-actin antibody (A5441) were obtained from Sigma (St. Louis, MO, USA). Protease inhibitor cocktail tablets (Complete, Mini^®^) were purchased from Roche (Mannheim, Germany). Bovine serum albumin (BSA), ethanol, xylene, and Ribonuclease (RNase) inhibitors were obtained from Fujifilm Wako (Osaka, Japan). ImmPACT™ DAB and VECTASTAIN Elite ABC kit were purchased from Vector Laboratories (Burlingame, CA, USA). Pacific blue (PB)-conjugated anti-mouse CD45 antibody (103126), brilliant violet (BV)-conjugated anti-mouse CD90.2 antibody (140319), fluorescein isothiocyanate (FITC)-conjugated anti-mouse lineage cocktail antibody (133301), phycoerythrin (PE)-conjugated anti-mouse CD278 antibody (107706), and PE/Cy7 conjugated anti-mouse CD3 antibody (100220) were purchased from BioLegend (San Diego, CA, USA). FITC-conjugated anti-mouse CD4 antibody (553651) and PE-conjugated anti-mouse CD25 antibody (50-5773-82) were purchased from BD Bioscience (San Jose, CA, USA). Nitrocellulose membranes (0.45 μm pore size) and 4–20% gradient gels were purchased from Bio-Rad (Hercules, CA, USA). dNTP mix, Random Primers, and CellTiter-Glo^®^ 2.0 were purchased from Promega (Madison, WI, USA). ReverTra Ace RT buffer was purchased from Toyobo (Osaka, Japan). A549 cells were kindly gifted from Dr. Takahisa Kuga (Setsunan University, Hirakata, Japan).

### 2.2. CNL Preparation

Preclinical development of the CNL has previously been described [23]. Briefly, lipids (distearoylphosphatidylcholine, dioleoylphosphatidylethanolamine, distearoylphosphatidylethanolamine-polyethylene glycol (PEG) 2000, PEG750-C_8_-ceramide with or without C_6_-ceramide to form the CNL or Ghost, respectively) were dissolved and mixed in chloroform, dried to a thin film under nitrogen, and then hydrated by addition of saline at 60 °C with sonication and vortexing. Lipid solutions were then extruded at 60 °C by passing through 100 nm polycarbonate filters. Size and charge were validated using a Malvern Zetasizer Nano (Malvern Panalytical, UK).

### 2.3. Sensitization and Challenges for Asthmatic Model

**Figure 1:**
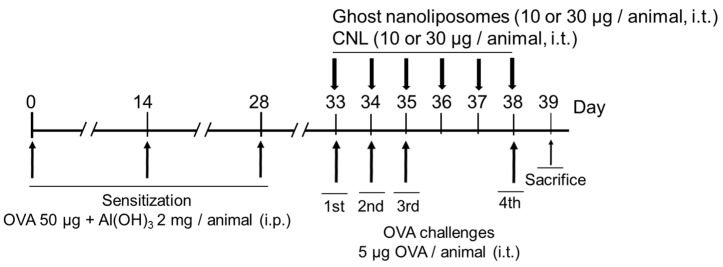
Animal experimental schedule. Mice were sensitized with OVA absorbed to Al(OH)_3_ on days 0, 14, and 28. Mice were intratracheally challenged with OVA-free PBS or OVA on days 33, 34, 35, and 38. At the indicated time points, Ghost or CNL (10 or 30 μg/mouse) were intratracheally administered prior to OVA challenges. Twenty-four hours later, mice were sacrificed.

The asthmatic model was established as previously reported [28]. Briefly, 5-week-old BALB/c mice (Japan SLC, Hamamatsu, Japan) were sensitized by i.p. injections with OVA adsorbed to Al(OH)_3_ at a dose of 50 μg of OVA/2 mg Al(OH)_3_/0.5 mL of saline/animal/time on days 0, 14 and 28. The sensitized mice were intratracheally challenged with OVA-free PBS or OVA (5 μg/25 µL/animal/time/day) on days 33, 34, 35, and 38. Intratracheal administration was conducted by insertion of a polyethylene tube from oral cavity under inhalation anesthesia with isoflurane as reported previously [29,30]. PBS, Ghost and CNL were intratracheally administered according to the schedule (Figure 1). Amounts of 10 or 30 μg CNL correspond to 1.4 or 4.2 μg C_6_-ceramide, respectively.

### 2.4. Ethics Statement

All animal studies were approved by the Experimental Animal Research Committee at Setsunan University (Hirakata, Japan).

### 2.5. Bronchoalveolar Lavage (BAL) Fluid Collection

BAL fluid analysis was performed as per previous studies [28,31]. Total leukocyte numbers were counted following hemolysis with ammonium–chloride–potassium (ACK) hemolysis buffer. Cells were settled on glass slides and stained with Diff-Quik solution (Sysmex International Reagent, Kobe, Japan). Eosinophils and neutrophils were observed by light microscope.

### 2.6. Immunohistochemistry

After the final challenge, mice were perfused with 20 mL PBS and 50 mL 10% formalin under anesthesia with pentobarbital and xylazine. Left lung lobes were isolated and cut into 3 sections. Formalin-fixed tissues were embedded in paraffin, and the 4-µm sections were stained using antibodies specific for MUC5AC, phospho-p38, and phospho-JNK along with the VECTASTAIN Elite ABC kit. Paraffin sections were deparaffinated with xylene and hydrophilized by 70% to 100% ethanol. After washing with water for 10 min, antigen retrieval was performed using HistoVT one according to the manufacture’s instruction. After washing with PBS containing 0.05% Tween 20 (PBS/0.05% Tween 20), the sections were treated with 3% hydrogen peroxide-methanol for 30 min. After washing with PBS/0.05% Tween 20, the sections were blocked with rabbit or goat normal serum for 30 min at room temperature. The sections were then treated with primary antibodies (MUC5AC, 1:100; phospho-p38, 1:100; and phospho-JNK 1:100) overnight at 4 °C. After washing with PBS/0.05% Tween 20, the sections were treated with biotin-conjugated anti-goat IgG antibody or anti-rabbit IgG antibody at room temperature for 30 min. After washing with PBS/0.05% Tween 20, lung sections were treated with avidin-HRP for 30 min at room temperature. After washing, the sections were incubated with ImmPACT™ DAB and counter-stained with hematoxylin. After staining, cell numbers were counted using the Hybrid Cell Count application (Keyence, Osaka, Japan).

### 2.7. Lung Histology

Histological examination was performed according to previous reports [28,31]. In brief, after the final OVA challenge, the left lobes of the lung were fixed with 10% neutral buffered formalin. Tissues were embedded in paraffin, and 4-μm sections were stained with Masson trichrome and periodic acid–Schiff (PAS). Histological changes were semi-quantitatively scored as described [31]. The degree of epithelial thickening was quantified using PAS-stained sections as follows: the bronchus (the diameter > 150 μm) was photographed, and then epithelial area (μm^2^) and basement membrane length were measured using version 1.42 Image J (NIH, Bethesda, MD, USA). The epithelial area was then divided by the basement membrane length. For quantification of the epithelial layer mucus accumulation, PAS-stained areas of the epithelium (μm^2^) were measured using Adobe Photoshop (Adobe Systems, San Jose, CA, USA) and version 1.42 Image J. The PAS-stained area was then divided by the length of the basement membrane. To assess subepithelial fibrosis, Masson trichrome staining was performed. Subepithelial fibrosis was assessed by the extent of the blue fibrotic area, and scored on a 7-point graded scale from 0 to 3 based on the distance that the blue fibrotic area extended underneath the basement membrane: zero, no blue area underneath the basement membrane; then 0.5, 1, 1.5, 2, 2.5, and 3, corresponding extensions of less than 15 μm, 15–30 μm, 30–45 μm, 45–60 μm, 60–75 μm, or more than 75 μm, respectively. All specimens were analyzed in a blind manner.

### 2.8. ELISA

ELISA was performed according to previous reports [28,31]. Briefly, the left lung lobes in T-PER containing protease inhibitor cocktails were homogenized with the polytron homogenizer. After centrifugation, supernatants were stored at −80 °C until measurement of type 2 cytokines (IL-4, IL-5, and IL-13) by ELISA. Type 2 cytokine concentrations were normalized relative to protein.

### 2.9. Analysis of ILC2, Treg, and Tr1 Cells

The left lungs were rinsed with PBS and tissues digested by 870 units/mL collagenase type I for 1 h at 37 °C. Cells were dispersed with a syringe then filtered through a 108 µm nylon mesh. After centrifugation, cells were treated with ACK lysis buffer to remove erythrocytes. The total leukocyte cell number was counted by trypan blue staining. ILC2s were defined as Lineage^-^ CD45^+^ CD278^+^ CD90.2^+^ ST2^+^ cells [28]. Treg cells were defined as CD4^+^ CD3^+^ CD25^+^ Foxp3^+^ cells [32]. Tr1 cells were defined as CD4^+^ CD3^+^ IL-10^+^ Foxp3^−^ cells [32] after the OVA stimulation.

For ILC2s analysis, lung cells were treated with an anti-CD16/32 (FcγRII/III) antibody to prevent the non-specific binding. After washing with PBS containing 2% FBS (PBS/2% FBS), cells were treated with PB-conjugated anti-mouse CD45 antibody, BV 510™-conjugated anti-mouse CD90.2 antibody, FITC-conjugated anti-mouse lineage antibody cocktail, PE-conjugated anti-mouse CD278 antibody, and allophycocyanin-conjugated anti-mouse ST2 antibody. After incubation for 20 min at 4 °C, cells were fixed with 4% paraformaldehyde for 15 h at 4 °C. After washing with PBS/2% FBS, the cells were analyzed using FACSAria™ Fusion (Becton Dickinson, CA, USA).

For Treg cell detection, lung cells were treated with anti-CD16/32 antibodies. After washing with PBS/2% FBS, cells were treated with FITC-conjugated anti-mouse CD4, PE/Cy7-conjugated anti-mouse CD3, and PE-conjugated anti-mouse CD25 antibodies. After incubation for 20 min at 4 °C, cells were washed with PBS/2% FBS and then incubated with Fixation/Permeabilization Concentrate for 12 h. After washing with permeabilization buffer, cells were stained with eFluor^®^660-conjugated anti-mouse Foxp3 antibody for 30 min at 4 °C. After washing with PBS/2% FBS, the stained cells were analyzed using FACSAria™ Fusion.

To detect Tr1 cells, lung cells were seeded on a plate. The cells were stimulated with 10 mg/mL OVA for 6 h at 37 °C followed by treatment with 2 µM monensin sodium salt. After washing with PBS/2% FBS, cells were treated with an anti-CD16/32 antibody. The cells were further stained with FITC-conjugated anti-mouse CD4 and PE/Cy7-conjugated anti-mouse CD3 antibodies for 20 min at 4 °C. After washing with PBS/2% FBS, cells were incubated with Fixation/Permeabilization Concentrate for 12 h. After washing with permeabilization buffer, the cells were stained with eFluor^®^660-conjugated anti-mouse Foxp3 antibody and PE-conjugated anti-mouse IL-10 antibody for 30 min at 4 °C. After washing with PBS/2% FBS, the cells were analyzed using the FACSAria™ Fusion.

### 2.10. Cell Culture

Human lung epithelial A549 cells were grown in DMEM supplemented with 10% FBS. The cells were maintained at <80% confluence under standard incubator conditions (humidified atmosphere, 95% air, 5% CO_2_, and 37 °C). Mycoplasma contamination was not observed in the cell lines.

### 2.11. Quantitative Real-Time PCR

RNAs were extracted from A549 cells using RNAqueous™-Micro Total RNA Isolation Kit (Thermo Fisher Scientific, Waltham, MA, USA). Reverse transcription was performed using ReverTra Ace^®^ with dNTP mix, and Random Primers, forming cDNA samples. Quantitative polymerase chain reaction was performed with a StepOne™ Real-Time PCR System (Thermo Fisher Scientific, Waltham, MA, USA). cDNA samples were mixed with Fast SYBR™ Green Master Mix, the forward and reverse primers for human MUC5AC (forward, 5′-CTGTGAAGGTGGCTGACCAAGA-3′; reverse, 5′-AAGGTGTAGTAGGTGCCGTCGAA-3′) or human glyceraldehyde-3-phosphate dehydrogenase (GAPDH) (forward, 5′-TGTTCGTCATGGGTGTGAAC-3′; 5′-ACTGTGGTCATGAGTCCTTCC-3′). The mixtures were heated at 95 °C for 20 s to activate Fast CYBR™. Denaturation (95 °C, 3 s), annealing and extension (60 °C, 30 s) were repeated for 40 cycles. The relative quantification of MUC5AC mRNA was calculated based on the 2^−ΔΔ^CT method.

### 2.12. Cell Viability Assay

A549 cells (4 × 10^4^ cells/well) were seeded on 96-well culture plate. Cell viability was determined using a CellTiter-Glo luminescent cell viability assay according to the manufacturer’s protocol.

### 2.13. Immunoblotting

A549 cells were washed with ice-cold PBS containing 10 mM EDTA and then lysed using sample buffer. Cell lysates were heated for 10 min at 98 °C. Proteins were then subjected to SDS-PAGE (4–20% gradient gels) and electrophoretically transferred to nitrocellulose membranes. Membranes were blocked with PBS/0.1% Tween 20 containing 5% nonfat dried milk, washed with PBS-T, and incubated with primary antibodies specific for phospho-EGFR (1 to 1,000), EGFR (1 to 1000), phospho-p38 (1 to 1000), p38α (1 to 1000), phospho-JNK (1 to 1000), JNK (1 to 1000), phospho-Akt (1 to 1000), Akt (1 to 1000), and β-actin (1 to 10,000) in PBS/0.1% Tween 20 containing 5% BSA. The blots were washed with PBS/0.1% Tween 20 and incubated with HRP-conjugated secondary antibodies in PBS/0.1% Tween 20 containing 5% nonfat dried milk. Proteins were detected using SuperSignal™ West Dura Extended Duration Substrate and ChemiDoc Imaging Systems (Bio-Rad, Hercules, CA, USA). After getting images, band intensities were quantified using Image Lab software version 6.1.0 (Bio-Rad, Hercules, CA, USA).

### 2.14. Statistical Analysis

Statistical analysis was performed with Graphpad Prism version 8. A one-way analysis of variance was performed. If significant differences were detected, individual differences were determined by Dunnett’s test. The unpaired *t*-test was used to compare two groups.

## 3. Results

**Figure 2:**
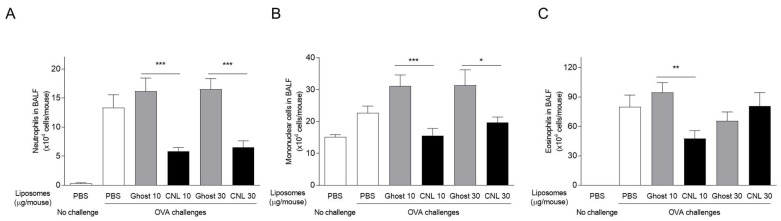
Effects of ceramide nanoliposomes (CNL) on leukocyte infiltration to BAL fluids in asthmatic mice. OVA-sensitized mice were challenged with OVA-free PBS or OVA. OVA-challenged asthmatic mice were treated with liposome-free PBS, ceramide-free ghost or CNL. The numbers of mononuclear cells (**A**), neutrophils (**B**), and eosinophils (**C**) in BAL fluids were determined by coulter counter and Diff-Quik staining. Data are shown as mean ± S.E. (*n* = 10–13). * *p* < 0.05, ** *p* < 0.01, *** *p* < 0.001 compared to the Ghost group.

The potential of liposomal ceramides, CNL, to act as an anti-asthmatic reagent was investigated. Ceramide-free ghost liposomes (Ghost) or CNL were intratracheally administered to mice (Figure 1). Firstly, airway inflammation was assessed by infiltration of inflammatory cells to BAL fluids. PBS/OVA-challenges increased the number of neutrophils, mononuclear cells and eosinophils in BAL fluids. At 10 or 30 μg/mouse CNL, OVA-induced infiltration of leukocytes such as neutrophils and mononuclear cells, was suppressed in comparison to the Ghost group (Figure 2A,B). In 10 μg/mouse CNL-treated mice, there was less eosinophil infiltration compared to the Ghost group (Figure 2C). The number of eosinophils in BAL fluids of 30 μg/mouse CNL group were comparable with the Ghost group.

**Figure 3:**
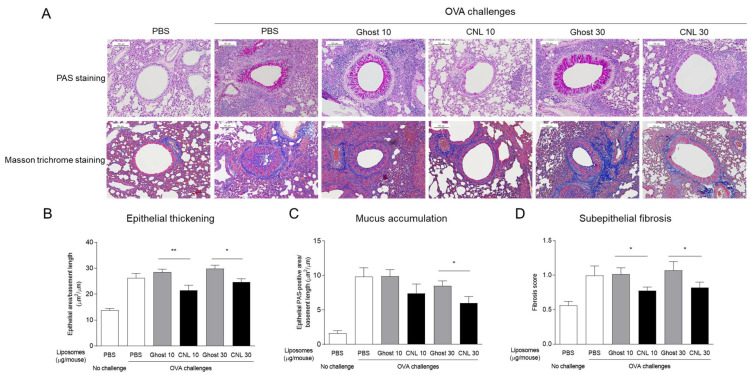
Effects of ceramide nanoliposomes (CNL) on airway remodeling. OVA-sensitized mice were challenged with OVA-free PBS or OVA. OVA-challenged asthmatic mice were treated with liposome-free PBS, ceramide-free Ghost or CNL. Lung tissues were subjected to histological analysis with PAS staining and Masson trichrome staining. Representative images of PAS and Masson trichrome staining are shown (**A**). In PAS staining, scores for epithelial thickening (**B**) and mucus accumulation (**C**) were determined. Fibrosis scores were quantified by Masson trichrome staining (**D**). Data are shown as mean ± S.E. (*n* = 10–13). * *p* < 0.05, ** *p* < 0.01 compared to the Ghost group.

Airway inflammatory responses are associated with airway histological changes, referred to as airway remodeling. Airway remodeling is a pathological feature of asthma, characterized by goblet cell hyperplasia-governed mucosa accumulation, bronchial epithelial thickening, subepithelial fibrosis, and hyperplasia of airway smooth muscle cells. To assess CNL effects on airway remodeling, lung tissues were subjected to PAS and Masson trichrome staining (Figure 3A). OVA-challenged mice displayed airway remodeling characterized by up-regulation of bronchial epithelial thickening, epithelial mucus accumulation, and subepithelial fibrosis (Figure 3B–D). CNL treatment significantly suppressed the progression of airway remodeling, though significant dose-dependent responses to CNL were not observed.

CD4^+^ regulatory T (Treg) cells are important in promoting immune tolerance to allergens and preventing allergic diseases [33]. Treg cells are classified into two subsets, Foxp3^+^ Treg cells or Foxp3^−^ Tr1 cells, which strongly produce the anti-inflammatory cytokine IL-10 [34]. In previous studies using the OVA asthmatic mouse model, Tr1 cells were shown to suppress infiltration of eosinophils and neutrophils into BAL fluids, airway remodeling, and IL-5 up-regulation [32]. We wondered if CNL affected immune tolerance. In response to OVA challenges, the number of Foxp3^+^ Treg and Tr1 cells in lung tissues increased (Table 1). However, CNL treatment had no significant effect on the number of cells, instead slightly decreasing numbers. Therefore, Treg subsets are unlikely to contribute to the anti-asthmatic effect of CNL.

ILC2s are implicated in the pathogenesis of type 2 inflammation-associated allergic asthma [35]. In response to airway epithelial cell-derived cytokines such as IL-33 and thymic stromal lymphopoietin, ILC2s produce large quantities of type 2 cytokines [28,36,37]. OVA challenges evoked significant increases in the number of lung ILC2s, though CNL treatment had no significant effect (Table 1).

**Figure 4:**
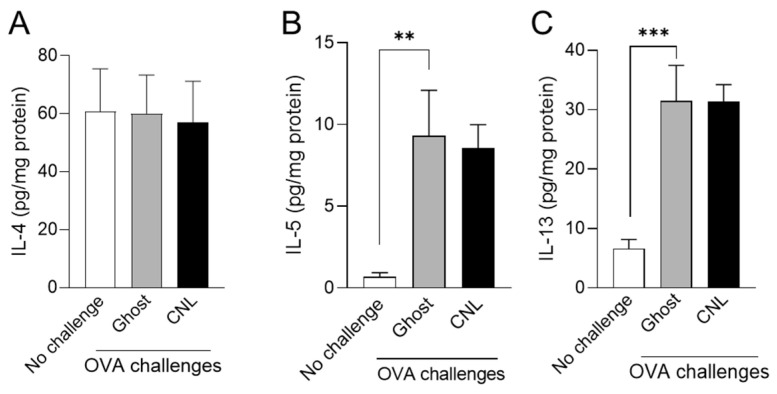
Effects of ceramide nanoliposomes (CNL) on the formation of type 2 cytokines in the lung. Asthmatic mice were treated with 30 µg/animal Ghost or CNL. Type 2 cytokines levels including IL-4 (**A**), IL-5 (**B**), and IL-13 (**C**) in the lung tissues were measured by ELISA. Concentration of type 2 cytokines were normalized to protein. Data are shown as mean ± S.E. (*n* = 12). ** *p* < 0.01, *** *p* < 0.001 compared to the Ghost group.

Type 2 cytokines, predominantly produced by ILC2, play key roles in airway remodeling and are emerging therapeutic targets [3]. We assessed CNL effects on the formation of IL-4, IL-5, and IL-13 in OVA-challenged lungs. In OVA-challenged mice, the formation of IL-5 and IL-13, but not IL-4, was significantly increased in lung homogenates, which was unsuppressed by CNL treatment (Figure 4). Those results suggest that CNL prevents mucus secretion and fibrosis in the lung epithelium without affecting levels of mucus secretion-stimulating cytokines IL-5 and IL-13.

**Figure 5:**
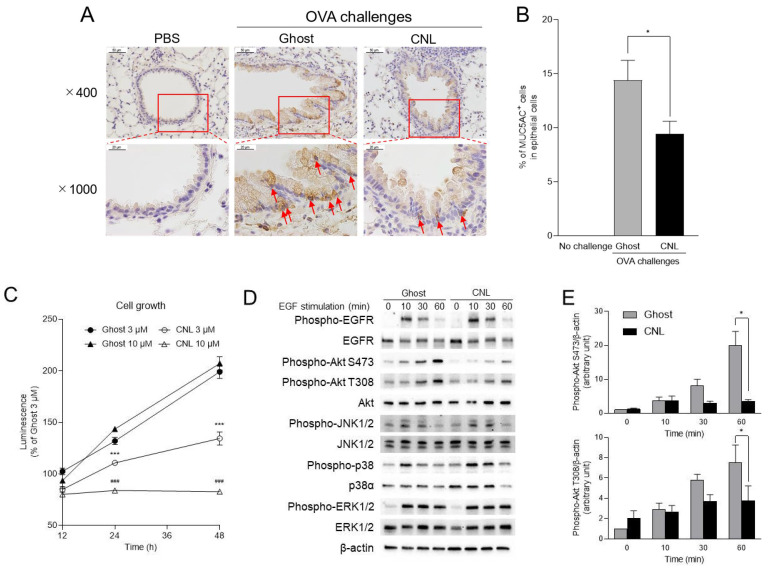
Effects of ceramide nanoliposomes (CNL) on goblet cell hyperplasia. (**A**,**B**) OVA-sensitized mice were challenged with OVA-free PBS or OVA. OVA-challenged asthmatic mice were treated with 30 µg/animal ceramide-free Ghost or CNL. Lung tissue sections were stained with MUC5AC antibody. Representative images of the immunohistochemistry on airway epithelia are shown and arrows show goblet cells (**A**). The ratios of goblet cells to total epithelial cells were determined (**B**). Data are shown as mean ± S.E. (*n* = 10). * *p* < 0.05 compared to the Ghost group. (**C**) Human lung epithelial A549 cells (4000 cells/well) were treated with the indicated concentration of Ghost or CNL for up to 48 h. Cell viability was determined using a CellTiter-Glo luminescent cell viability assay. Data are shown as mean ± S.E. (*n* = 4). Dose- and time-matched statistical analysis were performed. *** *p* < 0.001 compared to the Ghost 3 µM group. ### *p* < 0.001 compared to the Ghost 10 µM group. (**D**) A549 cells were treated with 10 µM Ghost or CNL for 6 h followed by stimulation with 100 ng/mL EGF for the indicated periods. Proteins were subjected to immunoblot analysis. Results are representative of three independent experiments. (**E**) Intensity of the immunoblots were quantified and phospho-Akt/β-actin values were calculated. The values are shown as percentages relative to Ghost 0 min group. Data are shown as mean ± S.E. (*n* = 3). * *p* < 0.05 compared to Ghost group.

Airway mucus is composed of mucus glycoproteins (mucins) including MUC5AC and MUC5B. Among them, MUC5AC accounts for approximately 90% of the mucin content of sputum [38]. Goblet cells in the bronchiolar epithelium predominantly secretes airway MUC5AC, with goblet cell hyperplasia and the associated mucus hypersecretion contributing to asthma airway remodeling [39,40,41]. In our asthma model, goblet cell hyperplasia, represented by an increased number of MUC5AC-positive goblet cells in the airway epithelium, was observed in OVA-challenged mice (Figure 5A). Importantly, CNL treatment significantly down-regulated MUC5AC-positive goblet cell numbers in airways to approximately 65% that observed after Ghost treatment, demonstrating the inhibitory effect of CNL on goblet cell hyperplasia (Figure 5B). As intratracheally administered CNL is logically thought to act on bronchiolar epithelial cells, airway goblet cell hyperplasia is a potential anti-asthmatic target for CNL. To model the effects of CNL on goblet cell hyperplasia, human epithelial cells recapitulating bronchial epithelial cells, including goblet cells, were employed. The epithelial cells cultured with 10% FBS showed time-dependent cell growth, which was significantly suppressed by CNL treatment (Figure 5C). Trypan blue exclusion assay revealed that 3 μM CNL had no significant effects on cell viability. Whereas 10 μM CNL, slightly but significantly, decreased the cell viability (Supplementary Figure S1). The short-chain C_6_-ceramide is a known apoptotic agent [42,43]. MCF-7 and HL-60 cells are sensitive to the apoptotic effects of C_6_-ceramide in the 3–10 µM range, whereas A549 cells are more resistant [44]. Moreover, liposomal C_6_-ceramide did not induce apoptosis of non-cancerous epithelial cells in vitro or in vivo in previous studies [21]. Therefore, it is conceivable that CNL with 3–10 µM C_6_-ceramide primarily limits growth and survival for lung epithelial A549 cells.

EGF is a potent growth factor for epithelial cells, including goblet cells [45,46]. To investigate the molecular mechanism(s) underlying CNL-suppressed cell growth, the effects of CNL on EGF signaling were examined using human lung epithelial A549 cells. Cells were stimulated with EGF for the indicated periods. Phosphorylation/activation of EGF receptors peaked at 10 min. Downstream kinases such as MAPKs and Akt were also phosphorylated/activated. Surprisingly, CNL had no significant effects on phosphorylation of EGFR and MAPKs including ERK1/2, JNK1/2, and p38 (Figure 5D and Supplementary Figure S2). Whereas CNL potently reduced Akt phosphorylation at the Ser473 and Thr308 residues (Figure 5D,E). This suggests selective CNL suppression of the EGF receptor-governed pathway of phosphatidylinositol-3 kinase (PI3K)-Akt, responsible for cell growth. Considering the goblet cell hyperplasia following the differentiation of airway epithelial cells to goblet cells in response to OVA-induced airway inflammation, CNL appears to target and suppress goblet cell hyperplasia and associated mucus hypersecretion.

**Figure 6:**
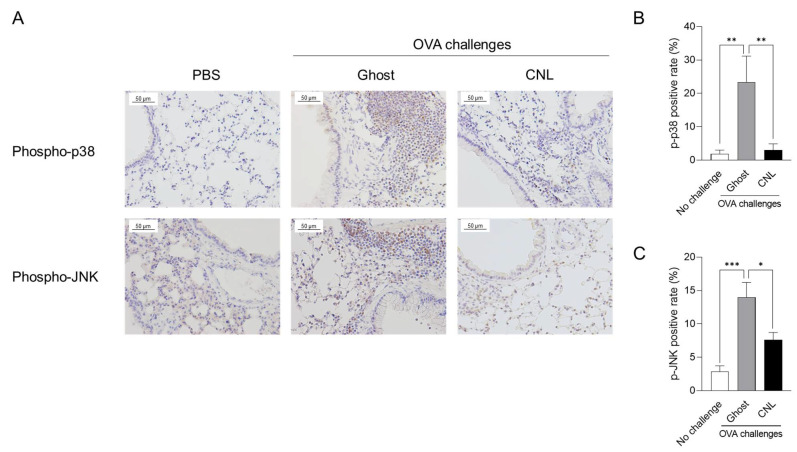
Effects of ceramide nanoliposomes (CNL) on p38 and JNK activation in asthmatic lungs. OVA-sensitized mice were challenged with OVA-free PBS or OVA. OVA-challenged asthmatic mice were treated with 30 µg/animal ceramide-free Ghost or CNL. Lungs isolated from mice were subjected to immunohistochemical analysis using antibodies specific to phospho-p38 and -JNK. Representative images of the immunohistochemistry are shown (**A**). (**B**) Ratio of phospho-p38 positive cells to total cells. Data are shown as mean ± S.E. (*n* = 4–5). ** *p* < 0.01 compared to the Ghost group. (**C**) Phospho-JNK positive cells ratio to total cells. Data are shown as mean ± S.E. (*n* = 8–9). * *p* < 0.05, *** *p* < 0.001 compared to the Ghost group.

The proinflammatory kinase p38 has emerged as a potential asthma therapeutic target [47,48,49]. In experimental models, p38 inhibition prevents allergen-induced goblet cell hyperplasia, mucus hypersecretion, and airway hyperresponsiveness [50,51]. In the lung tissues of OVA-challenged mice, the subepithelial regions where infiltrated leukocytes were observed, were positively stained with antibodies specific for phospho/active-p38, but the epithelial cells showed negative-staining (Figure 6A). CNL treatment drastically suppressed p38 activation (Figure 6B).

JNK is also a proinflammatory kinase associated with asthma promotion. JNK activation is implicated in airway epithelial cell differentiation into goblet cells, increased mucus production, and epithelial cell proliferation [52]. As with evaluating p38 activation, the subepithelial regions, excluding epithelial cells, were stained with antibodies specific for phospho/active-JNK1/2 in Ghost-treated asthmatic mice (Figure 6C). The CNL treatment suppressed JNK activation by about 50% (Figure 6D).

## 4. Discussion

Insufficient ceramide biosynthesis is a risk factor in non-allergic asthma [9,53]. In the present study, we demonstrated, in a murine model for the first time, the anti-asthmatic effects of intratracheal ceramide administration using nanoliposomal delivery. This mitigated the airway remodeling corresponding to irreversible pathological airway changes. Of the anti-asthmatic effects, noteworthy features of CNL treatment includes the inhibition of both mucosal accumulation and goblet cell hyperplasia in the airways. Mechanistically, CNL was thought to limit cell growth by inactivating EGF-governed AKT pathways, but not MAPK pathways. These findings support ceramide-based anti-inflammatory therapies for novel asthma treatments.

The pathophysiological roles of ceramides in the lung remains poorly understood. Biochemical studies in mouse lungs identified ceramide species compositions and the gene expression profile of ceramide synthases (CERSes) [54]. Very-long-chain C_24_-ceramide is a major species, and the loss of the CerS2 gene, which is responsible for very-long-chain ceramide biosynthesis, leads to significant airway flow obstruction, inflammation, increased lung volume, along with decreased very-long-chain ceramide and increased long-chain ceramide (C_16_-ceramide). Though the physiological roles for distinct ceramide species remain elusive, CERS2-governed biosynthesis and homeostasis of ceramide species are crucial for lung physiology.

Interestingly, Izawa et al. demonstrated that extracellular ceramides interacting with CD300f, an immune inhibitory receptor, limits OVA-induced chronic airway inflammation, characterized by the accumulation of inflammatory granulocytes and goblet cell hyperplasia [20], implicating ceramides as anti-asthmatic lipids. Interaction of CNL-derived ceramides with CD300f might contribute to anti-asthmatic effects of CNL.

In a house dust-mite-challenged asthma mouse model, lung ceramides were increased along with allergic response. Pharmacological inhibition of sphingolipid biosynthesis by intraperitoneal administration of myriocin or fumonisin B1 prevented reactive-oxygen species formation and apoptosis induction in lung tissues, as well as neutrophil recruitment to the lungs in mice [55]. In these studies, the effects of sphingolipid biosynthesis inhibition on asthmatic pathogenesis, such as airway remodeling and airway hyperresponsiveness, were not tested. Moreover, myriocin inhibits SPT, presumably suppressing biosynthesis of all sphingolipids, including the proinflammatory lipid sphingosine-1-phosphate. Sphingosine-1-phosphate and its generating enzyme, sphingosine kinase, are implicated in airway hyperresponsiveness and airway remodeling in asthma [56,57,58]. The molecular mechanisms underlying the inhibitory effects of myriocin on asthmatic inflammation appear complicated. Further extensive studies are needed to clarify the pathobiological roles of sphingolipid metabolism and metabolites in allergic asthma.

It is postulated that bronchial epithelial cells may take up CNL carrying C_6_-ceramide that is subsequently metabolized to sphingosine-1-phosphate. As intratracheal administration of CNL had substantial anti-asthmatic effects, ceramide action appears predominant in airways. Nevertheless, extensive CNL pharmacodynamic analysis is necessary.

Goblet cells are generally sparse in normal lung tissue and goblet cell hyperplasia is evoked by type 2 inflammation [59,60]. CNL is unlikely to significantly affect type 2 cytokine formation in asthmatic lung tissues (Figure 4). However, CNL acted as a potent inhibitor in goblet cell hyperplasia in asthmatic lung airways (Figure 5), which suggests that CNL targets asthmatic lung epithelia.

EGFR and IL-13 are believed to stimulate bronchial epithelial cells, including Clara and ciliated cells, to differentiate into goblet cells through the action of SPDEF and FoxA2 [39,61,62]. As CNL was reported to prevent embryonic stem cell differentiation [63], whether CNL suppresses goblet cell differentiation and growth needs to be tested in future studies. Our findings provide insights to establish novel molecular bases to understand the molecular pharmacological roles of CNL in inflammation-governed goblet cell differentiation and hyperplasia. In addition to asthma, goblet-cell hyperplasia is also a critical pathological feature of hypersecretory airway diseases, including chronic obstructive pulmonary disease and cystic fibrosis. Thus, CNL-based therapeutics may be effective against those lung diseases.

The PI3K-Akt pathway is proposed as a target for the growth-suppressing functions of ceramides [64,65]. Akt is activated by PI3K on the plasma membrane [66,67] and inactivated/dephosphorylated by Ser/Thr protein phosphatase PP2A [68]. Ceramides are potent inhibitory lipids toward PI3K, consequently preventing Akt activation [69]. Consistent with our results, C_6_-ceramide treatment selectively suppressed EGF activation of Akt at residues Thr308 and Ser473, but not ERK1/2 in epithelial ovarian cancer cells. Ceramide-activated PP2A is responsible for dephosphorylating Akt [64] and may be involved in the CNL inactivation of Akt. Various ceramide activities coordinately inactivate EGFR-governed PI3K-Akt signaling, which may be the mechanism behind the therapeutic effects of CNL on asthmatic lung epithelia.

Corticosteroid insensitivity is a clinical feature of severe asthma and p38 activation is involved in cellular corticosteroid insensitivity [48,70,71]. Suppression of p38 by CNL may reverse glucocorticoid resistance and improve the anti-inflammatory effects of glucocorticoids. Our future studies will determine therapeutic effectiveness of CNL in severe asthma model.

## 5. Conclusions

In conclusion, CNL, in an asthmatic model, was shown to have anti-inflammatory properties therapeutically effective for inhibition of airway remodeling. These novel findings shed light on potential therapeutic strategies for asthma through ceramide-based anti-inflammatory therapies as a novel class of asthma treatment.

## Figures and Tables

**Table 1:** Effects of ceramide nanoliposomes (CNL) on the number of ILC2, Treg, and Tr1 cells in lung tissues. OVA-sensitized mice were challenged with OVA-free PBS or OVA. OVA-challenged asthmatic mice were treated with 30 µg/animal ceramide-free Ghost or CNL. Cell numbers of ILC2, Treg, and Tr1 cells isolated from lungs were determined. Data are shown as mean ± S.E.

Cell Type (× 10^5^ Cells/Lung)	No OVA Challenge	OVA Challenge + Ghost	OVA Challenge + CNL
ILC2	1.67 ± 0.21 (*n* = 6)	7.13 ± 0.85 (*n* = 6)	8.15 ± 0.81 (*n* = 4)
Treg	1.25 ± 0.34 (*n* = 4)	6.67 ± 2.69 (*n* = 4)	3.74 ± 1.31 (*n* = 4)
Tr1	0.49 ± 0.10 (*n* = 4)	5.74 ± 0.83 (*n* = 4)	3.74 ± 0.80 (*n* = 4)

## Data Availability

Not applicable.

## Supplementary Materials

The following supporting information can be downloaded at: https://www.mdpi.com/article/10.3390/cells12040591/s1, Figure S1: Effects of CNL on cell viability in human lung epithelial A549 cells, Figure S2: Effects of CNL on EGF signaling in human lung epithelial A549 cells.
