# Supplementary material for: Ceramide Nanoliposomes as Potential Therapeutic Reagents for Asthma
Source: Cells. 2023 Feb 11;12(4):591. doi: 10.3390/cells12040591 (PMC9954069; doi:10.3390/cells12040591)
Supplement: Supplementary file 1 [file cells-12-00591-s001.zip › cells-2133388-supplementary.pdf]

Supplementary Figure 1

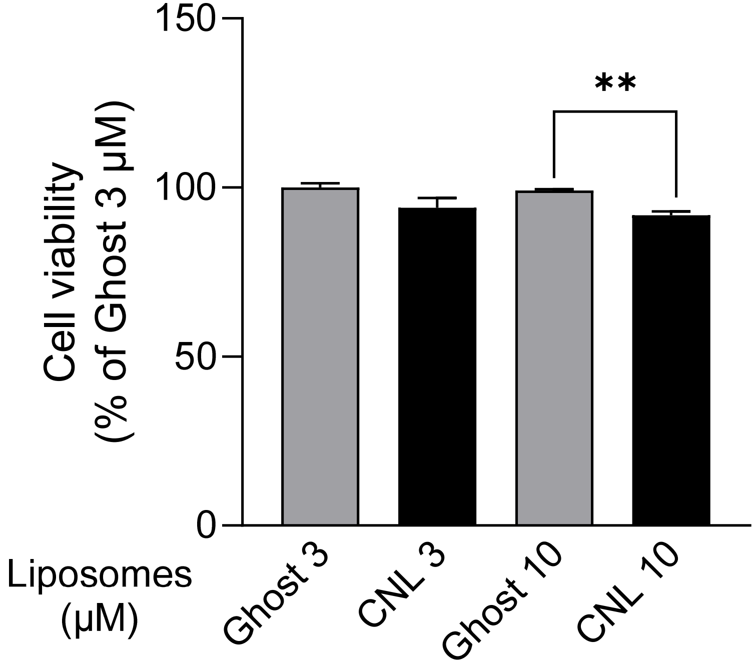

Effects of CNL on cell viability in human lung epithelial A549 cells
A549 cells plated in 6-well plates were treated with 3 or 10 μM Ghost or CNL for 24 h and then cell death was determined by trypan blue viability assay. Cells were suspended in PBS. A 1:1 dilution of the cell suspension was made using 0.4% trypan blue and loaded into a hemocytometer. Cell viability was immediately assessed. The data represent mean ± S.E. of three values. ** $p < 0.01$ compared to the Ghost 10 μM group.

Supplementary Figure 2

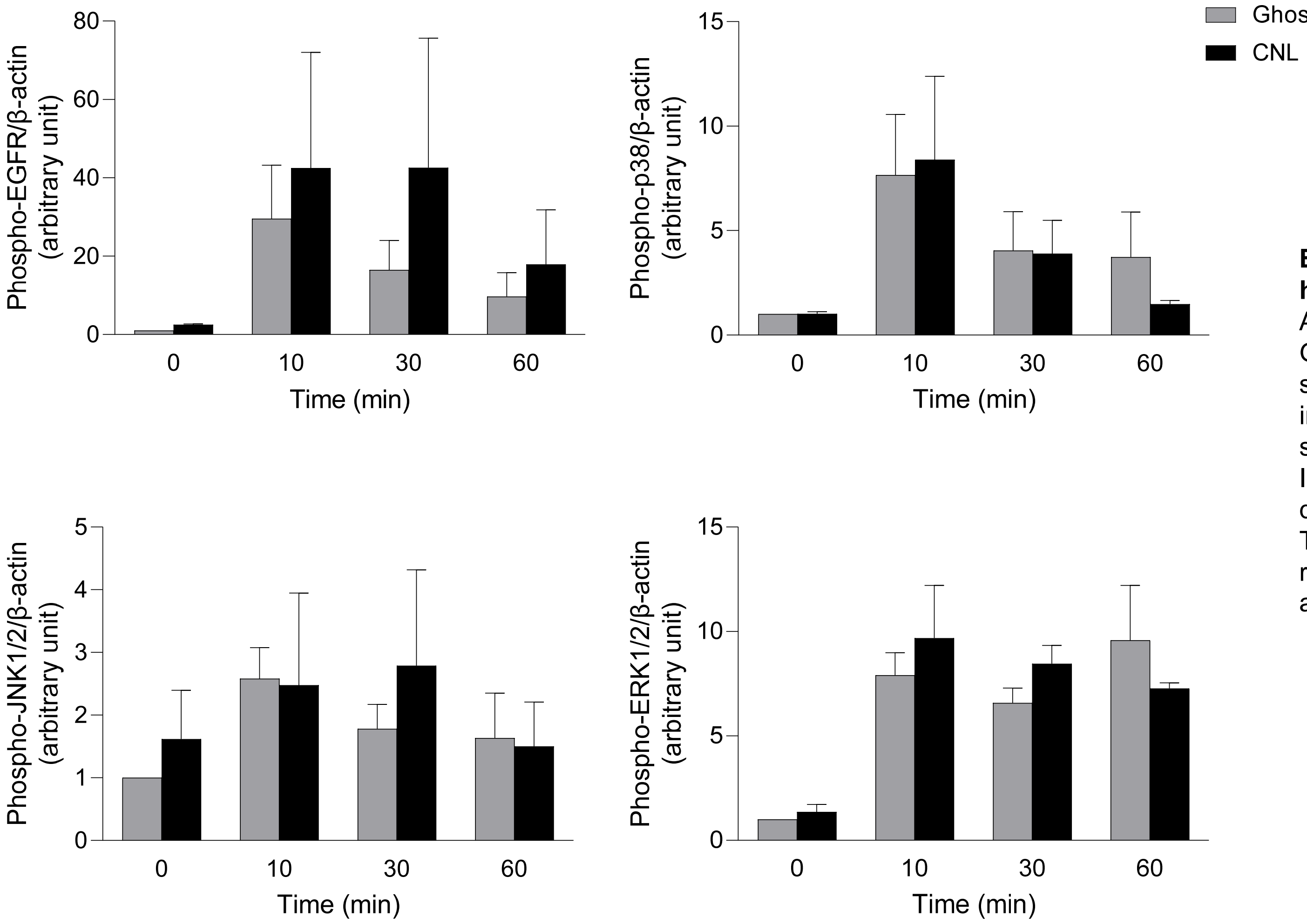

Effects of CNL on EGF signaling in human lung epithelial A549 cells

A549 cells were treated with 10 μM Ghost or CNL for 6 h followed by stimulation with 100 ng/ml EGF for the indicated periods. Proteins were subjected to immunoblot analysis. Intensity of the immunoblots were quantified and normalized with β-actin. The values are shown as percentages relative to Ghost 0 min group. Data are shown as mean ± S.E. (n=3).
